# Azithromycin-Induced Thrombocytopenia: A Rare Etiology of Drug-Induced Immune Thrombocytopenia

**DOI:** 10.1155/2019/6109831

**Published:** 2019-07-08

**Authors:** Muhammad Umer Butt, Ahmad Jabri, Samy Claude Elayi

**Affiliations:** ^1^Cardiology Department, Case Western Reserve University/MetroHealth Medical Center, Cleveland, OH, USA; ^2^Internal Medicine Department, Akron General Cleveland Clinic, Akron, OH, USA; ^3^Cardiac Electrophysiology Department, University of Florida Jacksonville Medical Center, Jacksonville, FL, USA

## Abstract

Drug-induced thrombocytopenia requires a high suspicion for diagnosis and a broad investigation to exclude other etiologies of low platelets. Cessation of the offending agent often results in recovery of platelet counts. Many medications are known to cause a degree of thrombocytopenia. We present a rare case of severe thrombocytopenia associated with administration of azithromycin.

## 1. Introduction

Medications that may cause thrombocytopenia continue to be identified in the literature. Drug-induced thrombocytopenia can result in severe, progressive reductions in platelet counts with resultant sequelae of bleeding [1–3]. In order to associate thrombocytopenia with a medication, recent exposure to the medication should be documented in addition to an investigation excluding all other causes of thrombocytopenia. Identification of drug-induced thrombocytopenia is important as the condition is reversible upon discontinuing the medicine [4, 5]. We present a rare case of azithromycin causing thrombocytopenia.

## 2. Case Presentation

A 25-year-old African American woman presented to the emergency department with a chief complaint of generalized nonpruritic maculopapular rash. The rash was initially confined to the right antecubital fossa but spread diffusely across the entire body over the course of 2 days. The patient had no other complaints and was otherwise in her usual state of health. The patient had started taking azithromycin 5 days prior to admission for a tooth extraction. She was not taking any other medication. There was no history of any recent illnesses or sick contacts. Vitals were within normal limits. Skin examination was significant for generalized petechiae and ecchymoses sparing her head and face. The patient did not have signs or symptoms of major bleeding.

Laboratory workup upon admission revealed an isolated thrombocytopenia with a platelet level of 2,000/*μ*L (normal range: 150–400 K/*µ*L). Repeat platelet count was 3,000/*μ*L, and the same value resulted when using a citrated tube. The hemoglobin was within the normal range at 12.3 mg/dl (normal range: 12.0 to 15.5 g/dL). Prothrombin time was increased with an elevated INR. A positive ANA and elevated ESR were found. Quantitative c-ANCA, p-ANCA, C3 Level, C4 Level, lupus anticoagulant, and dsDNA antibodies were measured and found to be within normal limits. Viral serology for hepatitis, HIV, CMV, and EBV was negative. Blood culture was negative for bacterial infection. A blood smear was performed and ruled out drug-induced thrombotic microangiopathy. Bone marrow core biopsy and aspirate smears showed increased megakaryocytes, myeloid cell hyperplasia, and left-shifted myeloid maturation. There were no sideroblasts or increase of blasts, lymphocytes, or plasma cells. Flow cytometry of bone marrow revealed no significant immunophenotypic abnormalities.

Azithromycin was discontinued. Dexamethasone 40 mg IV daily and intravenous immunoglobulin (IVIG) therapy were administered. Platelet counts improved to 17,000/*μ*L over the following 10 days. Gradually, over three months, platelet counts returned within the normal range. Drug-dependent anti-platelet antibody testing was not done during admission as the patient's platelet count began to slowly improve after stopping the azithromycin.

## 3. Discussion

Drug-induced immune thrombocytopenia is attributed to decreased platelet production secondary to myelosuppression or accelerated platelet destruction secondary to an immune response [1]. Drug-induced thrombocytopenia can present with asymptomatic thrombocytopenia, hemolytic-uremic syndrome (HUS), or thrombotic thrombocytopenia purpura (TTP) in extreme cases [2]. Drug-induced thrombocytopenia results in severely decreased platelet counts, often less than 20,000/*μ*L [3]. In most circumstances, a week's duration is often needed for the offending drug to induce thrombocytopenia. Discontinuing the medication can result in resolution of thrombocytopenia in as little as 4–8 days, although less commonly within weeks. [2].

It is quite challenging to establish the temporal association of thrombocytopenia and administration of medication especially when a patient is taking multiple medications. Clinical criteria were developed by Hackett et al. for the diagnosis of drug-induced thrombocytopenia which includes (1) thrombocytopenia occurring after drug usage and discontinuing the drug results in resolution of thrombocytopenia, (2) all possible causes of thrombocytopenia were excluded, (3) no other drug was started prior to the thrombocytopenia or other medications except for the offending drug were continued with no effect on the platelet count, and (4) reexposure of the offending drug results in recurrence of thrombocytopenia [4]. Meeting all four criteria makes the diagnosis of drug-induced thrombocytopenia definite. When criteria 1, 2, and 3 are met, as in our case, drug-induced thrombocytopenia is considered probable. Meeting criteria 1 suggests possible diagnosis of drug-induced thrombocytopenia [4]. The diagnosis is clinical and usually based upon thorough testing that rules out other causes of thrombocytopenia including hypersplenism, lymphoproliferative disorders, infections, mononucleosis, and autoimmune disorders and documenting resolution of symptoms after discontinuation of the suspected medication. Recurrent thrombocytopenia after reexposure to medication further supports the diagnosis [4, 5]. Platelet-reactive antibody testing can be used to assist in diagnosis; however, this testing is limited by availability and an uncertain sensitivity [2]. The management of drug-induced thrombocytopenia relies on first stopping the offending agent and then managing symptoms of bleeding. Platelet transfusions are initiated in presence of apparent hemorrhage [2, 6]. Since idiopathic thrombocytopenic purpura (ITP) is often hard to exclude, corticosteroids and/or IVIG are often initiated [3]. However, no benefit has been associated with immune suppression using corticosteroids in drug-induced thrombocytopenia [2].

We presented a rare case of azithromycin-induced thrombocytopenia. While macrolide-induced thrombocytopenia has been reported with clarithromycin [7, 8], it has been rarely reported with azithromycin. [9] In the reported case by Azharuddin et al., the azithromycin-induced thrombocytopenia resolved within three days despite the 48–72 hour half-life of azithromycin [9]. Our case has a prolonged course of thrombocytopenia that is not explained by the half-life of azithromycin. It was previously postulated that patients produce drug-dependent and drug-independent antibodies after exposure to an offending medication [10, 11]. Drug-independent antibodies, also known as autoantibodies, are most often transient; however, they can persist for a long period of time. This may have contributed to the prolonged recovery phase for our patient [12]. Since the risk of drug-induced thrombocytopenia is low when starting new medication, excluding other causes of thrombocytopenia is necessary [2]. However, given the extensive list of drugs implicated, physicians should have high suspicion when a patient presents with severe, isolated thrombocytopenia within two weeks of a new drug exposure [13].

## 4. Conclusion

Drug-induced thrombocytopenia is a clinical diagnosis after exclusion of other etiologies of thrombocytopenia. Azithromycin is a rare cause of severe thrombocytopenia that has been highlighted in our case. Our patient experienced resolution of thrombocytopenia after stopping azithromycin, although she had a delayed recovery time.
